# Performance of rK39-based immunochromatographic rapid diagnostic test for serodiagnosis of visceral leishmaniasis using whole blood, serum and oral fluid

**DOI:** 10.1371/journal.pone.0230610

**Published:** 2020-04-02

**Authors:** Maria Carmen Arroyo Sanchez, Beatriz Julieta Celeste, José Angelo Lauletta Lindoso, Mahyumi Fujimori, Roque Pacheco de Almeida, Carlos Magno Castelo Branco Fortaleza, Angelita Fernandes Druzian, Ana Priscila Freitas Lemos, Vanessa Campos Andrade de Melo, Anamaria Mello Miranda Paniago, Igor Thiago Queiroz, Hiro Goto

**Affiliations:** 1 Laboratório de Soroepidemiologia e Imunobiologia, Instituto de Medicina Tropical da Faculdade de Medicina, Universidade de São Paulo, SP, Brazil; 2 Departamento de Doenças Infecciosas e Parasitárias, Faculdade de Medicina, Universidade de São Paulo, SP, Brazil; 3 Instituto de Infectologia Emílio Ribas, Secretaria de Estado da Saúde, São Paulo, SP, Brazil; 4 Departamento de Medicina Interna e Patologia, Hospital Universitário/EBSERH, Universidade Federal de Sergipe, Aracaju, SE, Brazil; 5 Departamento de Doenças Tropicais e Diagnóstico por Imagem, Universidade Estadual Paulista Júlio de Mesquita Filho, Botucatu, SP, Brazil; 6 Hospital Universitário Maria Aparecida Pedrossian, Universidade Federal de Mato Grosso do Sul, Campo Grande, MS, Brazil; 7 Hospital Universitário Onofre Lopes, Universidade Federal do Rio Grande do Norte, Natal, RN, Brazil; 8 Hospital Giselda Trigueiro, Secretaria Estadual da Segurança Pública, Natal, RN, Brazil; 9 Faculdade de Medicina, Universidade Federal de Mato Grosso do Sul, Campo Grande, MS, Brazil; 10 Departamento de Medicina Preventiva, Faculdade de Medicina, Universidade de São Paulo, SP, Brazil; University of Liverpool, UNITED KINGDOM

## Abstract

**Background:**

The development of rK39-based immunochromatographic rapid diagnostic tests represents an important advance for serodiagnosis of visceral leishmaniasis, being cheap and easy to use at the point of care (POC). Although the use of rK39 have considerably improved the sensitivity and specificity of serological tests compared with total antigens, great variability in sensitivity and specificity was reported. This study aimed at the evaluation of “Kalazar Detect™ Rapid Test, Whole Blood” (Kalazar Detect RDT) for Visceral Leishmaniasis (VL) diagnosis using oral fluid, whole blood and serum specimens collected at different endemic areas of VL of Brazil.

**Methodology:**

To evaluate Kalazar Detect RDT, oral fluid, whole blood and serum specimens from 128 VL patients, 85 healthy individuals, 22 patients with possible cross-reactivity diseases and 20 VL/aids coinfected patients were collected and assayed at the POC.

**Principal findings and conclusions:**

The performance of Kalazar Detect RDT in whole blood and serum was similar; however, using oral fluid, the sensitivity was low. Particularly in samples from the city of Natal, Rio Grande do Norte state in Northeastern Brazil, we observed low sensitivity, 80.0% (95% CI: 62.7–90.5), using whole blood and serum, and poor sensitivity, 43.3% (95% CI: 27.4–60.8) with oral fluid. Those values were much lower than in the other regions, where sensitivity ranged from 92.7–96.3% in whole blood and serum, and 80.0–88.9% in oral fluid. Besides, in VL/aids coinfected patients, lower sensitivity was achieved compared with VL patients. In samples from Natal, the sensitivity was 0.0% (95% CI: 0.0–49.0) and 25.0% (95% CI: 4.6–69.9), using oral fluid and serum/whole blood, respectively; in samples from the other regions, the sensitivity ranged from 40.0–63.6% and 80.0–81.8%, respectively. As for specificity, high values were observed across the fluids, 100.0% (95% CI: 96.5–100.0) in whole blood, 96.3% (95% CI: 90.8–98.5) in serum, and 95.3% (95% CI: 89.5–98.0) in oral fluid; across localities, specificity ranged from 85.7–100.0%. Serum samples sent by the collaborating centers to Instituto de Medicina Tropical (n = 250) were tested by Kalazar Detect RDT, Direct Agglutination Test, Indirect immunofluorescence assay, Enzyme-linked immunosorbent assay, and IT-Leish® RDT. The regional difference in the performance of rK39-based RDT and lower sensitivity in *Leishmania*/HIV coinfected patients raise concern on the routine use of these products for the diagnosis of VL.

## Introduction

Visceral leishmaniasis (VL) is a disease caused by a protozoan of the genus *Leishmania*, the species *Leishmania* (*Leishmania*) *donovani* prevalent in the Indian subcontinent and East Africa, and *L*. (*L*.) *infantum* in other parts of Asia and Africa, Europe and New World [1–3]. *L*. (*L*.) *infantum* infection is transmitted by the bite of sandflies belonging to *Phlebotomus* spp, and dogs are the main peridomestic reservoir [4]. The World Health Organization (WHO) estimates that 50 000 to 90 000 new cases of VL occur worldwide each year and in 2017 more than 90% of new cases occurred in 10 countries: Bangladesh, Brazil, China, Ethiopia, India, Kenya, Somalia, South Sudan and Sudan [5]. In the Americas, 90% of VL cases occur in Brazil, where it is detected in 22 states and the Federal District, with 4,103 cases and 8.8% lethality in 2017 [6–8].

Parasitological techniques remain the gold standard for the diagnosis of leishmaniasis. They are highly specific, but with low sensitivity, reaching 52–85% in bone marrow aspirates that are the usual source for VL diagnosis [9]. The sensitivity may be higher when spleen aspirate is obtained (93.1–98.7%) [2], but in Brazil, it is not done for ethical reasons. Since bone marrow aspirates further require hematologist for evaluation not always available or accessible at the point of care (POC) or nearby, serological tests arise as alternative tools for the diagnosis of *Leishmania* infections [10]. As the POC in endemic areas does not always have a laboratory structure, among serological assays, the choice appoints to those that do not require laboratory equipment and trained technicians such as direct agglutination test (DAT) and rapid diagnostic test (RDT).

In 1998, the use of the recombinant K39 (rK39) antigen, derived from a 39 amino acid repeat encoded by a kinesin-like protein-encoding gene of *L*. *chagasi* [11], inserted onto immunochromatography platforms, commonly referred to as RDTs, represented an advance for the serological diagnosis and control of VL in Indian subcontinent [12]. RDTs made easy the field applicability of serological diagnosis because they do not require equipment, the procedure is simple, and the results are obtained within some minutes upon sample application on a chromatographic strip. These characteristics expedited the diagnosis of VL leading to a more appropriate and earlier treatment with a consequent reduction in lethality [13,14] Although recombinant antigens have considerably improved the sensitivity and specificity of serological tests compared with total antigens, great variability in sensitivity (82–100%) and specificity (86–100%) was reported [13,15–17]. Further data derived from the use of RDT for the diagnosis of VL in different studies and different regions showed quite different sensitivity of the test that impelled the WHO to coordinate a global comparative evaluation of four commercial immunochromatographic RDTs for VL that involved nine laboratories in East Africa, the Indian subcontinent and Latin America. This study showed a difference in the quality of commercially available RDTs and a regional difference in sensitivity of the same RDT. Generally, the RDTs sensitivities were lower in East Africa and Brazil compared with the Indian subcontinent [10]

In endemic areas worldwide, not only in Brazil, since the POC for VL has precarious infrastructures for laboratory diagnosis, we aimed in the present study at the evaluation of RDTs using different body fluid besides plasma, such as whole blood and oral fluid which are more easily obtained. Further due to the variability observed in RDT performance among regions [10], even when involving the same *Leishmania* species, this aspect was also considered in the present study.

In the present study, we observed good performance of Kalazar Detect RDT using whole blood and serum but poor with oral fluid. Besides, in VL/aids coinfected patients, lower sensitivity was achieved in relation to Visceral Leishmaniasis patients, regardless of the sample source. We also observed a regional difference in performance in some cases, reaching sensitivity lower than 90% that raises concern on the use of rK39-based RDT in the routine of VL diagnosis.

## Materials and methods

### Ethical statement

This project received ethical approval from the Research Ethics Committee of the Institute of Tropical Medicine and the Ethics Committee of the School of Medicine, University of São Paulo, on 07/03/2012 (approval number 490/11). According to Resolution No. 466 of 12/12/2012 (National Health Council, Brazil), all individuals enrolled in the study and/or their legal guardians (if the participant was a child) were informed of the objectives and voluntarily signed a written 'Informed Consent Form'.

### Study protocol

For this study, sampling was prospectively collected in collaborating centers from the Brazilian leishmaniasis endemic area and the non-endemic municipality of Sao Paulo, between 2012 and 2013. Fingertip blood, saliva, and blood without anticoagulant for serum separation were obtained from VL patients, healthy asymptomatic individuals, patients with potentially cross-reactive diseases, and VL/aids coinfected patients. The three fluids were assayed at the sites of collection by “Kalazar Detect™ Rapid Test, Whole Blood” (Kalazar Detect RDT), and serum samples were sent to Instituto de Medicina Tropical da Faculdade de Medicina/USP (IMT/FMUSP) to be tested by Kalazar Detect RDT and other serological assays.

### Sample size calculation

Assuming that the sensitivity of Kalazar Detect RDT was 95% [18] and considering a 95% confidence interval (95% CI) of ± 5%, the sample size needed was at least 73 VL patients. As for specificity, at least 84 controls were required, assuming 98% specificity [18] and considering a 95% CI of ± 3% [19].

### Study subjects

Two hundred forty subjects were enrolled in the four collaborating centers from Brazilian leishmaniasis endemic area and 15 from the non-endemic municipality of Sao Paulo (S1 Fig): 1. Universidade Federal de Mato Grosso do Sul (Campo Grande/MS), 2. Universidade Estadual de Sao Paulo–UNESP (Botucatu/SP), 3. Universidade Federal de Sergipe (Aracaju/SE), 4. Hospital Giselda Trigueiro–SESAP (Natal/RN), 5. Instituto de Infectologia Emilio Ribas (Sao Paulo/SP) (Table 1). The subjects enrolled were distributed in four groups:

Group I—VL patients—consisted of 128 patients from endemic area, with a combination of two of the following symptoms or signs, fever, hepatomegaly and/or splenomegaly, cytopenia but excluding those with confirmed diagnosis of HIV or a disease with possibility of cross-reactivity, as cutaneous leishmaniasis, tuberculosis, malaria, systemic mycoses, leprosy and Chagas disease). VL disease was confirmed by parasitological exam in a bone marrow sample by microscopy/culture [9] and when it was not possible to do the parasitological exam, by positive Direct Agglutination Test (DAT) [10]. These patients were from Campo Grande/MS, Bauru/SP, Aracaju/SE, and Natal/RN.

**Table 1:** Demographic data of the study subjects. Number of samples from different patients and controls, and site of collection.

Subjects	Sex		Age (yr)		
	M	F	Median	Min-max	Kruskal-Wallis (p)
**Visceral Leishmaniasis (n = 128)**	86	39	25.5	1–90	<0.001
Campo Grande (n = 27)	19	8	43 a	1–90	
Bauru (n = 16)	9	7	16.5	1–59	
Aracaju (n = 55)	35	20	9.5	1–68	
Natal (n = 30)	23	4	33	15–56	
**Asymptomatic control (n = 85)**	46	39	30	2–67	<0.001
Campo Grande (n = 31)	20	11	34	4–67	
Bauru (n = 7)	0	7	32	29–35	
Aracaju (n = 14)	6	8	9.5 b	2–33	
Natal (n = 33)	20	13	29	16–60	
**Potentially cross-reactive control (n = 22)**	-	-	-	-	
Campo Grande (n = 7)	4	3	43	25–74	
Sao Paulo (n = 15)	-	-	-	-	
**Visceral Leishmaniasis/aids (n = 20)**	12	8	36	20–53	0.667
Campo Grande (n = 11)	8	3	37	20–53	
Bauru (n = 5)	3	2	43	23–53	
Natal (n = 4)	1	3	31	21–46	

n–Number of samples.

M–Male. F–Female.

Min-max–minimum-maximum.

*a*–p<0.001 (Kruskal-Wallis) in relation to Bauru and Aracaju.

*b*–p<0.001 in relation to Bauru, Campo Grande, and Natal.

Group II—asymptomatic controls—consisted of 85 individuals considered healthy by their assessment and/or clinical exam, matched by age and sex with VL patients, living in an area with transmission of *Leishmania* infection, who stayed asymptomatic for VL for six months follow-up and who was negative by DAT. These individuals were from Campo Grande/MS, Bauru/SP, Aracaju/SE, and Natal/RN.

Group III—controls with possible cross-reactivity—consisted of 22 patients with a confirmed diagnosis of Chagas disease (N = 6), paracoccidioidomycosis (N = 7), cutaneous leishmaniasis (N = 7) and tuberculosis (N = 2), and who were DAT negative. These patients were from Campo Grande/MS and Sao Paulo/SP.

Group IV–Among VL samples, 20 were later identified as being from co-infected VL/aids patients and, although the number was small, we analyzed the influence of this co-infection on the performance of the RDT. These patients were from Campo Grande/MS, Bauru/SP, and Natal/RN.

All samples were randomly assayed to ensure that the technicians performed a blinded analysis.

### Laboratory tests performed at the site of sample collection, at the POC

To standardize the results among the centers, the coordinator center (IMT/FMUSP) sent SOPs (standard operating procedure) of “Coordinator Assignments”, “Collection, sample identification and processing and Kalazar Detect RDT execution” and “Kalazar Detect™ Rapid Test, Whole Blood–reading and recording the results”. We also sent forms, “First medical record—collection and recording of clinical and epidemiological data”, “Record of sample identification codes and clinical and epidemiological data”, “Technician 1 result sheet”, “Technician 2 result sheet” and “Record of Kalazar Detect RDT results”.

“Kalazar Detect™ Rapid Test, Whole Blood” (Kalazar Detect RDT) (Inbios International, Seattle, WA) was performed, following manufacturer instructions, in whole blood, oral fluid, and serum, at the collection sites. Ten microliters of the specimen were dispensed onto the device by micropipette, and the buffer was applied using the dropper provided. All samples (Table 1) were tested with lot 1 of RDT, and 25% of the samples were tested simultaneously with a second lot (Lot 2) to assess lot‐to‐lot reproducibility. Two technicians performed independent readings and recorded the results on separate standardized forms. Technician 1 read at the minimum reading time (15 minutes) and Technician 2 after 30 minutes from the first reading. The test result was considered positive when both the control line and test line were present and negative when only the control line was seen. In the absence of the control line, the test was considered invalid and repeated twice. To standardize the Kalazar Detect RDT reading at the POC (S2 Fig), control line and test line color intensities were scored as strong positive (3), positive (2), weak positive (1) and faint (0.5). The absence of test lines was recorded as negative (0).

After RDT assay at the POC, all samples from VL patients were sent to Instituto de Medicina Tropical de Sao Paulo/USP to be tested, except five (four from Aracaju and one from Bauru) that had problem in storing.

### Laboratory tests performed at Instituto de Medicina Tropical da Faculdade de Medicina/USP (IMT)

In addition to Kalazar Detect ^TM^ RDT performed as in the POC, all serum samples sent to IMT (n = 250) were tested by Direct Agglutination Test–DAT, Indirect immunofluorescence assay–IFA, Enzyme-linked immunosorbent assay–ELISA and IT-Leish ® RDT (Bio-Rad Laboratories).

DAT based on *L*. *infantum*-derived antigen kit produced at Prince Leopold Institute of Tropical Medicine, Amsterdam, The Netherlands, was performed using serum samples, according to Harith and collaborators [20] Prior to use, DAT *cut-off* was calculated using a panel of 56 samples from parasitologically confirmed cases of VL and 48 healthy controls from VL endemic area. Using a cut-off of ≥1:3,200, DAT presented 98.2% (90.4–99.9) sensitivity (95%CI) and 100.0% (92.6–100.0) specificity. For the study, 260 samples were two-fold diluted from 1:100 through 1:102,400.

IFA based on *L*. *major*-like promastigotes was performed in serum samples, according to Guimaraes and collaborators [21] ELISA based on a total alkaline extract of *L*. *major*-like promastigotes (MHOM/BR/71/49) was performed in serum samples, according to Celeste and collaborators [22]. IT-Leish RDT was performed according to manufacturer instructions, following the same protocol used for Kalazar Detect RDT, for qualitative and quantitative evaluation of the reaction and readings involving two Technicians.

### Data management and analysis

Sensitivity, specificity, positive and negative likelihood ratios and accuracy were calculated for the RDT with 95% confidence intervals (CI), using the results from the Technician 1 reading at 15 minutes. For comparison of sensitivity and specificity, McNemar chi-square was used, after matching controls and VL cases, considering the DAT/parasite result. Differences between proportions were tested for significance using the Fisher exact probability test or chi-square test. Titers and band intensity color were compared by Friedman test (paired samples), Kruskal-Wallis (three or more groups) or Mann-Whitney Rank Sum Test (two groups). Inter-reader and inter-lot reliabilities (reproducibility) were calculated for the results expressed as positive and negative using equations from Fleiss and collaborators [23] According to Landis and Koch [24], the *kappa* (κ) coefficient associated with the relative strength of agreement was assigned as 1.00–0.81 almost perfect, 0.80–0.61 substantial, 0.60–0.41 moderate, 0.40–0.21 fair, and 0.20–0.00 slight agreement; < 0 no agreement.

The likelihood ratio of a positive test (LR+) is the proportion of positive results obtained among those with the disease (sensitivity) divided by the proportion of positive results obtained among those without the disease (1-specificity). LR+ >1 indicates evidence for the disease. The likelihood ratio of a negative test (LR–) is the proportion of negative results obtained among those with the disease (1-sensitivity) divided by the proportion of negative results obtained among those without the disease (specificity) [25,26]. LR–<1 indicates evidence for the absence of disease. LR+ >10 and LR–<0.1 indicates strong evidence for the disease or the absence of disease, respectively [27,28]. Intuitively, the higher the LR+, the greater the evidence for disease and the smaller the LR–the lesser the association with the presence of the disease [29]. When specificity is 100%, LR+ was not calculated.

Positive and negative predictive values (PPV and NPV) were not calculated because they depend mathematically on prevalence of the disease [19,30] and our sampling was collected based on a previous diagnosis (convenience sampling) and do not represent the real prevalence of VL in the settings studied.

Statistical analyses were performed using the GraphPad Prism version 5.00 for Windows (GraphPad Software, San Diego California USA, www.graphpad.com), GraphPad QuickCalcs (©2018 GraphPad Software, https://www.graphpad.com/quickcalcs/catMenu/), SigmaStat for Windows version 3.5 (©2006 Systat Software, Inc.) and MedCalc Statistical software version 19.1 (©2019 MedCalc Software bvba, Ostend, Belgium, https://www.medcalc.org/calc/diagnostic_test.php).

## Results

### Direct agglutination test–DAT

VL cases and controls were categorized by DAT as either positive with low, medium, or high antibody titer or negative [10] Three control samples that were DAT positive and two VL samples that were DAT negative without a parasitological exam were not selected for the study (S1 Fig).

Table 2 shows the results obtained with the 250 samples that met the inclusion criteria. All asymptomatic control samples and potentially cross-reactive samples were DAT negative (≤1:1,600). Three case samples were DAT negative, two from Leishmania/HIV coinfected patients and one from a VL patient. DAT sensitivity was 99.2% in VL patients and 90.0% in VL/aids patients (Fisher's exact test, p = 0.0507).

**Table 2:** Median titers obtained in DAT for VL, VL/aids and control samples.

Locality	VL		AC		CR		VL/aids	
	median	min-max	median	min-max	median	min-max	median	min-max
**Campo Grande**	102,400	3,200–409,600	100	100–1,600	100	100–1,600	12,800	100–409,600
**Bauru**	51,200	25,600–409,600	100	100–100	-	-	51,200	25,600–102,400
**Aracaju**	153,600	6,400–409,600	100	100–200	-	-	-	-
**Natal**	204,800	200–409,600	100	100–400	-	-	3,200 a	1,600–25,600
**Sao Paulo**	-	-	-	-	100	100–100	-	-
**Total**	**102,400**	**200–409,600**	**100**	**100–1,600**	**100**	**100–1,600**	**25,600 b**	**100–409,600**

DAT–direct agglutination test.

VL–visceral leishmaniasis.

AC–asymptomatic control.

CR–potentially cross-reactive control.

VL/aids–coinfected patients.

min-max–minimum-maximum.

*a*–p = 0.0056 (Mann-Whitney Rank Sum Test) in relation to VL patients from Natal.

*b*–p = 0.0004 (Mann-Whitney Rank Sum Test) in relation to total VL patients.

Among localities, no significant difference was observed in VL samples (Kruskal-Wallis Analysis of Variance, p = 0.0736), in VL/aids samples (Kruskal-Wallis Analysis of Variance, p = 0.0712) and in potentially cross-reactive samples (Mann-Whitney Rank Sum Test, p = 0.068). A significant difference was observed in asymptomatic controls (Kruskal-Wallis Analysis of Variance, p = 0.0282), but Dunn's Multiple Comparison Test does not detect the difference between pairs of localities.

Comparing VL and VL/aids DAT titers, no significant difference was observed in Campo Grande (Mann-Whitney Rank Sum Test, p = 0.1792) and Bauru (Mann-Whitney Rank Sum Test, p = 0.9637). However, a significant difference was observed between VL and VL/aids DAT titers in Natal (Mann-Whitney Rank Sum Test, p = 0.0056) and considering all localities (Mann-Whitney Rank Sum Test, p = 0.0004).

### “Kalazar Detect™ Rapid Test, Whole Blood” performed at the site of sample collection, at the POC

Tables 3–7 and S1 Fig show the results of RDT performed in whole blood (WB), oral fluid (OF) and serum (SE) samples at the POC. We present the reading of technician 1 with lot 1. These results refer to the samples in Table 1.

**Table 3:** Sensitivity (%) and 95% confidence intervals (95% CI) of the RDT performed in oral fluid, serum and whole blood samples from VL patients, according to the collection site.

Locality (n)	Sensitivity% (n)		
	95% CI		
	Oral fluid	Serum	Whole blood
**Campo Grande (27)**	88.9 (24)	96.3 (26)	96.3 (26)
	71.9–96.1	81.7–99.3	81.7–99.3
**Bauru (16)**	81.2 (13)	93.7 (15)	93.7 (15)
	57.0–93.4	71.7–98.9	71.7–98.9
**Aracaju (55)**	80.0 (44)	90.9 (50)	92.7 (51)
	67.6–88.4	80.4–96.0	82.7–97.1
**Natal (30)**	43.3 (13) a c	80.0 (24)	80.0 (24)
	27.4–60.8	62.7–90.5	62.7–90.5
**Total (128)**	**73.4 (94) b**	**89.8 (115)**	**90.6 (116)**
	**65.2–80.3**	**83.4–94.0**	**84.3–94.6**

n–Number of samples.

*a*–p = 0.004 and.

*b*–p<0.001 (McNemar’s test) in relation to serum and whole blood.

*c*–p = 0.0003 (chi-square test) in relation to Campo Grande, Bauru and Aracaju.

**Table 4:** Comparing RDT test line intensity in oral fluid, serum and whole blood samples collected from patients with VL, according to the collection site.

Locality (n)	Median ^c^		
	Oral fluid	Serum	Whole blood
**Campo Grande (27)**	2.0 a	3.0	3.0
**Bauru (16)**	1.0 b	3.0	2.5
**Aracaju (55)**	1.0 a	3.0	3.0
**Natal (30)**	0.0 a	2.5	3.0
**Total (128)**	**1.0 a**	**3.0**	**3.0**

n–number of samples.

*a*–p<0.0001 (Friedman test for paired samples) in relation to whole blood and serum.

*b*–p = 0.0003 (Friedman test for paired samples) in relation to serum.

*c*–Median of the test line intensities scores: strong positive (3), positive (2), weak positive (1) and faint (0.5); no test line (0).

**Table 5:** Sensitivity (%) and 95% confidence intervals (95% CI) of the RDT performed in oral fluid, serum and whole blood samples from VL/aids patients, according to the collection site.

Locality (n)	Sensitivity% (n)		
	95% CI		
	Oral fluid	Serum	Whole blood
**Campo Grande (11)**	63.6 (7)	81.8 (9)	81.8 (9)
	35.4–84.8	52.3–94.9	52.3–94.9
**Bauru (5)**	40.0 (2)	80.0 (4)	80.0 (4)
	11.8–76.9	37.6–96.4	37.6–96.4
**Natal (4)**	0.0 (0)	25.0 (1)	25.0 (1)
	0.0–49.0	4.6–69.9	4.6–69.9
**Total (20)**	**45.0 (9)**	**70.0 (14)**	**70.0 (14)**
	**25.8–65.8**	**48.1–85.4**	**48.1–85.4**

n–number of samples.

No difference across localities (chi-square test, p>0.05) or fluids (McNemar’s test, p>0.05.

**Table 6:** Comparing RDT test line intensity in oral fluid, serum and whole blood samples from VL/aids patients, according to the collection site.

Locality (n)	Median ^c^			p
	Oral fluid	Serum	Whole blood	
**Campo Grande (11)**	1.0	2.0 a	1.0	0.0273
**Bauru (5)**	2.0	2.0	2.0	0.1821
**Natal (4)**	0.0	0.0	0.0	0.4306
**Total (20)**	**0.5**	**1.5 b**	**1.0**	**0.0031**

n–number of samples.

*a*–p = 0.0273 and.

*b*–p = 0.0031 (Friedman test for paired samples) in relation to whole blood and oral fluid.

*c*–Median of the test line intensities scores: strong positive (3), positive (2), weak positive (1) and faint (0.5); no test line (0).

**Table 7:** Specificity (%) and 95% confidence intervals (95% CI) of the RDT performed in oral fluid, serum and whole blood samples from asymptomatic and potential cross-reactive controls, according to the collection site.

Locality (n)	Specificity% (n)		
	95% CI		
	Oral fluid	Serum	Whole blood
**Campo Grande (38)**	92.1 (35)	94.7 (36)	100.0 (38)
	79.2–97.3	82.7–98.5	90.8–100.0
**Bauru (7)**	100.0 (7)	100.0 (7)	100.0 (7)
	64.6–100.0	64.6–100.0	64.6–100.0
**Aracaju (14)**	92.9 (13)	85.7 (12)	100.0 (14)
	68.5–98.7	60.1–96.0	78.5–100.0
**Natal (33)**	100.0 (33)	100.0 (33)	100.0 (33)
	89.6–100.0	89.6–100.0	89.6–100.0
**Sao Paulo (15)**	93.3 (14)	100.0 (15)	100.0 (15)
	70.2–98.8	79.6–100.0	79.6–100.0
**Total (107)**	**95.3 (102)**	**96.3 (103)**	**100.0 (107)**
	**89.5–98.0**	**90.8–98.5**	**96.5–100.0**

n–Number of samples.

No difference across localities (chi-square test, p>0.05) or fluids (McNemar’s test, p>0.05).

In samples from VL patients (Tables 3 and S1 and S1 Fig), it was observed a lower sensitivity with oral fluid compared to whole blood and serum in Natal (McNemar’s test, p = 0.004) and when the samples of all the centers were considered together (McNemar’s test, p<0.001). Comparing the performance of each fluid (whole blood, oral fluid and serum) at the sites of collection, lower sensitivity was obtained in Natal, with oral fluid, (chi-square test, p = 0.0003). Furthermore, in Natal whole blood and serum yielded an 80.0% sensitivity, much lower than in the other localities (92.7% to 96.3%).

Taking into account VL patients from all localities, RDT test line intensity (S2 Fig) was significantly weaker using oral fluid compared to serum and whole blood (Friedman test for paired samples, p = 0.0003 and p<0.0001) (Table 4); no difference was observed across the localities (Kruskal-Wallis test, p>0.05). In asymptomatic controls and potentially cross-reactive controls, medians of test line intensity were zero using the three fluids (Friedman test for paired samples, p>0.05).

In samples from VL/aids patients (Table 5 and S1 Fig), the sensitivity obtained was not significantly different across the localities and for the three fluids. The diagnostic performance of whole blood was very similar to that of serum, which provides one more attribute for using Kalazar Detect RDT at the POC.

Considering VL/aids patients (Table 6), RDT test line intensity (S2 Fig) was significantly different among fluids in Campo Grande (Friedman test for paired samples, p = 0.0273) and when the samples of localities were considered all together (Friedman test for paired samples, p = 0.0031). No difference was observed across the localities (Kruskal-Wallis test, p>0.05).

In controls, no difference was observed in the specificity of the RDT across the localities and fluids (Table 7 and S1 Fig).

Considering VL and control samples (n = 220, excluding coinfected VL/aids and other diseases patients from Sao Paulo), Kalazar Detect RDT achieved the highest accuracy (95% CI) using serum and whole blood in relation to oral fluid, respectively, 92.3% (87.9–95.4), 94.5% (90.7–97.1) and 82.7% (77.1–87.5). Positive likelihood ratios (LR+) were >10 across the localities and for all fluids, except in Aracaju for serum sample (6.36; 95%CI: 1.76–26.02). The best negative likelihood ratio (LR-) was achieved with whole blood (0.09; 95%CI: 0.05–0.16). On the other hand, LR- were >0.1 across the localities for oral fluid and higher in Natal, not only with oral fluid (0.57; 95%CI: 0.41–0.77) but also with serum and whole blood (0.20; 95%CI: 0.10–0.41) (S1 Table).

**Lot-to-lot and interobserver variability.** The agreement of Kalazar Detect results between readers, using lot 1 (S2 Table) and lot-to-lot agreement (S3 Table) was almost perfect [24].

### Tests performed at IMT

Tables 8–10 and S3 Fig present the results of Kalazar Detect^TM^–Rapid Diagnostic RDT, IT-Leish® RDT, indirect immunofluorescence assay (IFA) and Enzyme-linked immunosorbent assay (ELISA) obtained assaying the serum (SE) samples sent to IMT. The results obtained with Kalazar Detect at the POC are also presented in the first column for comparison.

**Table 8:** Sensitivity (%) and 95% confidence intervals (95% CI) of Kalazar Detect performed at the point of care (POC) and IT-Leish and other tests performed at IMT, in serum samples collected from patients with VL, according to the collection site.

Locality (n)	Sensitivity % (n)				
	CI 95%				
	KD-POC	KD-IMT	IT-Leish	IFA	ELISA
**Campo Grande (27)**	96.3 (26)	96.3 (26)	96.3 (26)	85.2 (23)	96.3 (26)
	81.7–99.3	81.7–99.3	81.7–99.3	67.5–94.4	81.7–99.3
**Bauru (15)**	93.3 (14)	93.3 (14)	93.3 (14)	60.0 (9)	93.3 (14)
	70.2–98.8	70.2–98.8	70.2–98.8	35.7–80.2	70.2–98.8
**Aracaju (52)**	94.2 (49)	86.5 (45)	98.1 (51) a	82.7 (43)	98.1 (51) a
	84.4–98.0	74.7–93.3	89.5–99.6	70.2–90.6	89.9–99.7
**Natal (30)**	80.0 (24)	80.0 (24)	86.7 (26)	83.3 (25)	86.7 (26)
	62.7–90.5	62.7–90.5	70.3–94.7	66.4–92.7	70.3–94.7
**Total (124)**	**91.1 (113)**	**87.9 (109)**	**94.4 (117) b**	**80.6 (100)**	**94.4 (117) b**
	**84.8–95.0**	**81.0–92.5**	**88.8–97.2**	**72.8–86.6**	**88.8–97.2**

n–number of samples.

KD-POC–Kalazar Detect performed at the point of care.

KD-IMT–Kalazar Detect processed at IMT.

IT-Leish–rK39 –RDT.

IFA–*L*. *major*-like based Indirect immunofluorescence assay.

ELISA–*L*. *major*-like based Enzyme-linked immunosorbent assay.

*a*–p = 0.041 and.

*b*–p = 0.013 (McNemar’s test) in relation to KD-IMT.

No difference across localities (chi-square test, p>0.05).

Concerning VL samples (Table 8 and S3 Fig and S4 Table), higher sensitivity was achieved in IT-Leish and ELISA (McNemar’s test, p = 0.041) in samples from Aracaju and the total samples (McNemar’s test, p = 0.013) in relation to Kalazar Detect performed at IMT (Kalazar Detect-IMT). Across localities, no significant difference was observed in any of the tests.

Regarding VL/aids samples, no difference across localities (chi-square test, p>0.05) or tests (McNemar’s test, p>0.05) was observed (Table 9 and S3 Fig).

**Table 9:** Sensitivity (%) and 95% confidence intervals (95% CI) of Kalazar Detect performed at the point of care (POC) and IT-Leish and other tests performed at IMT, in serum samples collected from patients with VL/aids, according to the collection site.

Locality (n)	Sensitivity % (n)				
	CI 95%				
	KD-POC	KD-IMT	IT-Leish	IFA	ELISA
**Campo Grande (11)**	81.8 (9)	63.6 (7)	72.7 (8)	72.7 (8)	81.8 (9)
	79.2–97.3	35.4–84.8	43.4–90.3	43.4–90.3	79.2–97.3
**Bauru (5)**	80.0 (4)	80.0 (4)	80.0 (4)	80.0 (4)	80.0 (4)
	37.6–96.4	37.6–96.4	37.6–96.4	37.6–96.4	37.6–96.4
**Natal (4)**	25.0 (1)	25.0 (1)	25.0 (1)	75.0 (3)	75.0 (3)
	4.56–70.0	4.6–69.9	4.6–69.9	30.1–95.4	30.1–95.4
**Total (20)**	**70.0 (14)**	**60.0 (12)**	**65.0 (13)**	**75.0 (15)**	**80.0 (16)**
	**48.1–85.5**	**38.7–78.1**	**43.3–81.9**	**53.1–88.8**	**58.4–91.9**

n–number of samples.

KD-POC–Kalazar Detect performed at the point of care.

KD-IMT–Kalazar Detect processed at IMT.

IT-Leish–rK39 –RDT.

IFA–*L*. *major*-like based Indirect immunofluorescence assay.

ELISA–*L*. *major*-like based Enzyme-linked immunosorbent assay.

No difference across localities (chi-square test, p>0.05) or tests (McNemar’s test, p>0.05).

Higher sensitivity was achieved in VL serum samples (Table 8) in relation to VL/aids (Table 9) in the total of samples using Kalazar Detect-POC (Fisher's exact test, p = 0.0155), Kalazar Detect-IMT (Fisher's exact test, p = 0.0046), IT-Leish (Fisher's exact test, p = 0.0007) and ELISA (Fisher's exact test, p = 0.0474). Higher sensitivity in VL samples was also obtained in Natal using Kalazar Detect-POC (Fisher's exact test, p = 0.0480), Kalazar Detect-IMT (Fisher's exact test, p = 0.0480) and IT-Leish (Fisher's exact test, p = 0.0211), and in Campo Grande using Kalazar Detect-IMT (Fisher's exact test, p = 0.0187) (S5 Table). Across localities, no significant difference was observed in Kalazar Detect-POC, Kalazar Detect-IMT, IT-Leish, IFA and ELISA sensitivities, in both VL (Table 8) and VL/aids samples (Table 9).

No difference was observed in specificity values obtained across different localities (chi-square, p>0.05). Lower specificity was achieved by ELISA in relation to Kalazar Detect-IMT, in samples from Campo Grande (McNemar’s test, p = 0.002), Aracaju (McNemar’s test, p = 0.041), Natal (McNemar’s test, p = 0.001) and Sao Paulo (McNemar’s test, p = 0.003), and in the total of samples (McNemar’s test, p<0.001) (Table 10 and S3 Fig).

**Table 10:** Specificity (%) and 95% confidence intervals (95% CI) of Kalazar Detect performed at the point of care (POC) and IT-Leish and other tests performed at IMT, in serum samples from asymptomatic and potential cross-reactive controls, according to the collection site.

Locality (n)	Specificity% (n)				
	CI 95%				
	KD-POC	KD-IMT	IT-Leish	IFA	ELISA
**Campo Grande (38)**	94.7 (36)	89.5 (34)	97.4 (37)	97.4 (37)	55.3 (21) a
	82.7–98.5	75.9–95.8	86.5–99.5	86.5–99.5	39.7–69.8
**Bauru (7)**	100.0 (7)	100.0 (7)	100.0 (7)	100.0 (7)	71.4 (5)
	64.6–100.0	64.6–100.0	64.6–100.0	64.6–100.0	35.9–91.8
**Aracaju (13)**	92.3 (12)	84.6 (11)	84.6 (11)	76.9 (10)	38.5 (5) b
	63.7–98.6	57.8–95.7	57.8–95.7	49.7–91.8	17.7–64.5
**Natal (33)**	100.0 (33)	97.0 (32)	100.0 (33)	84.8 (28)	60.6 (20) c
	89.6–100.0	84.7–99.5	89.6–100.0	69.1–93.3	43.7–75.3
**Sao Paulo (15)**	100.0 (15)	100.0 (15)	100.0 (15)	73.3 (11)	26.7 (4) d
	79.6–100.0	79.6–100.0	79.6–100.0	48.0–89.1	10.9–51.9
**Total (106)**	**97.2 (103)**	**93.4 (99)**	**97.2 (103)**	**87.7 (93)**	**51.2 (55) e**
	**92.0–99.0**	**87.0–96.8**	**92.0–99.0**	**80.1–92.7**	**42.5–61.2**

n–number of samples.

KD-POC–Kalazar Detect performed at point of care.

KD-IMT–Kalazar Detect processed at IMT.

IT-Leish–rK39 –RDT.

IFA–*L*. *major*-like based Indirect immunofluorescence assay.

ELISA–*L*. *major*-like based Enzyme-linked immunosorbent assay.

No difference across localities (chi-square, p>0.05).

*a*–p = 0.002,

*b*–p = 0.041,

*c*–p = 0.001,

*d*–p = 0.003,

*e*–p<0.001 (McNemar’s test) in relation to KD-IMT.

It is important to point out that the low specificity of IFA and ELISA observed among the Sao Paulo controls is due to the cases of cutaneous leishmaniasis and Chagas disease that react with *L*. *major*-like promastigotes (IFA) and *L*. *major*-like total extract, antigen used in ELISA (S6 Table).

Considering VL and control samples (n = 215, excluding coinfected VL/aids and other diseases patients from Sao Palo), IT-Leish RDT, KD-POC and KD-IMT achieved the highest accuracy (95% CI), respectively, 95.3% (91.6–97.7), 93.5% (89.3–96.4) and 89.8% (84.9–93.5) in relation to IFA and ELISA, respectively, 84.6% (79.1–89.2) and 78.1% (72.0–83.5). IT-Leish RDT and KD-POC showed the highest LR+ (28.62; 95%CI: 9.40–87.16 and 27.64; 95%CI: 9.07–84.23, respectively) and the lowest LR- (0.06; 95%CI: 0.03–0.12 and 0.09; 95%CI: 0.05–0.16, respectively). Across the localities and in the total of VL and control samples, ELISA showed LR+ <3 and IFA showed LR- >0.1. In Natal, LR- were >0.1 in all tests (S4 Table).

## Discussion

In the present study, we aimed at the evaluation of the performance of rK39-RDT, Kalazar Detect RDT, for VL diagnosis using different body fluids as source and using samples collected in different regions of Brazil. The performance of Kalazar Detect RDT using whole blood and serum was good but with oral fluid, the sensitivity was poor. Particularly with samples from the city of Natal, Rio Grande do Norte state, Northeastern Brazil, we observed sensitivity below 90%, using whole blood and serum and lower using oral fluid. Due to the difference in performance, apparently regional, we evaluated the results, using another rK39-RDT, the IT-Leish RDT, and the same serum samples. The performance of IT-Leish RDT was slightly better than that of Kalazar Detect, but the lower sensitivity with samples from Natal was confirmed. In VL/aids coinfected patients, lower sensitivity was achieved in relation to VL patients, mainly in Natal.

Overall, whole blood and serum showed high sensitivity in VL patients and high specificity in asymptomatic and potential cross-reactive controls. However, in VL/aids coinfected patients, low sensitivity was achieved. These results corroborate other studies [10,31–33], in which sensitivity ranged from 83–95%, in VL patients, and from 61–67% in HIV/aids coinfected patients and the specificity ranged from 90–100%.

In contrast, in VL patients, oral fluid showed poor sensitivity (McNemar’s test, p<0.001) and lower median scores of the test line intensity with whole blood and serum, not only in the total of samples but also in Campo Grande, Bauru, Aracaju and Natal. Our results are in agreement with studies carried out in India, where Vaish and collaborators evaluated rK39-RDT and rK-39-ELISA in VL patients and obtained a sensitivity of 100% and around 80% in both tests, using serum and saliva samples, respectively [34]. In another study, Mohapatra and collaborators compared serum, urine, and saliva samples of VL cases using rK39-RDT. Saliva was positive in 84% of cases, and serum and urine in 100% [35]. da Silva and collaborators concluded that saliva is not an appropriate material for diagnosing VL using rK39-RDT, as they obtained 58.6% sensitivity in VL cases [36]. On the other hand, in Tunisian VL patients, a sensitivity of 100% was achieved with oral fluid in biotin-streptavidin ELISA using rK39 [37] The reason for this difference may be attributable to the assay format, parasite diversity and /or level of antibody response that may be related to the severity of the disease since the Tunisian patients included in that study were children admitted at the hospital.

A point that must be emphasized is the performance of samples from VL patients collected in Natal. Although the difference has been significant only with oral fluid (chi-square test, p = 0.0003), the sensitivity using whole blood and serum from Natal resulted around 10–15% below that obtained using the samples from the other regions and remained below 90%. According to the recommendations of Boelaert and collaborators, an ideal VL diagnostic test for case detection should have a sensitivity of ≥ 95% and specificity ≥ 98% [18]The unsatisfactory performance observed in Natal, regardless of the fluid used, can be evidenced by the high values of LR- were in Natal, not only with oral fluid (0.57; 95%CI: 0.41–0.77) but also with serum and whole blood (0.20; 95%CI: 0.10–0.41).

Differences in rapid test performance may be due to parasite diversity and /or differences in antibody levels, which may be associated with age, immune response, and nutritional status of the patient [10]. However, in our study, the proportion between men and women in Natal was not different from the other regions studied (chi-square test, p = 0.261). Regarding the median age group in each locality, a difference was observed only in Aracaju, where a lower median was observed.

The 250 serum samples sent by the sites of collection to IMT (besides Kalazar Detect) were submitted to additional tests including DAT as an estimate of total antibody reactivity [10], due to the good sensitivity and specificity reported van Griensven and Diro [38], IT-Leish, IFA and ELISA. Although DAT sensitivity was not statistically different in VL patients (99.2%) and VL/aids patients (90.0%), (Fisher's exact test, p = 0.0507), DAT median titers were lower in VL/aids patients in relation to VL patients (Mann-Whitney Rank Sum Test, p = 0.0056) in Natal and in the total of samples (Mann-Whitney Rank Sum Test, p = 0.0004).

Assaying sera from VL and VL/aids patients, this study evaluated two different RDT (Kalazar Detect and IT-Leish) and in house IFA and ELISA and obtained sensitivity of 87.9%, 94.4%, 80.6% and 94.4%, respectively, in VL patients, and 60.0%, 65.0%, 75.0% and 80.0%, respectively, in VL/aids patients. Although both RDT tests are rK39-based immunochromatographic tests, lower sensitivity was observed using Kalazar Detect-IMT in relation to IT-Leish and ELISA (McNemar’s test, p = 0.013). Our results are in accordance to the evaluation led by the World Health Organization [10] in which IT-Leish sensitivity (92.0%) was significantly higher than that of Kalazar Detect (84.7%) in samples from Brazil. Across localities, no significant difference was observed (chi-square test, p>0.05), although in Natal all tests showed sensitivity below 90%.

Concerning the different tests applied, aids status in VL patients, and the locality studied, very low sensitivity (25%) was observed in coinfected patients from Natal using Kalazar Detect (POC and IMT) and IT-Leish. These results corroborate those of other authors [32,39] that found low sensitivity in coinfected patients. When considering the localities altogether except for IFA and DAT, all tests had lower sensitivity in coinfected patients. The higher sensitivity of DAT in relation to Kalazar Detect-IMT both in VL (McNemar’s test, p<0.001) and in coinfected patients (McNemar’s test, p = 0.041) is in line with. van Griensven and Diro [38]

As for specificity, both Kalazar Detect-IMT and IT-Leish presented high values, 93.4% and 97.2%, respectively, that corroborate other authors [10,33].

IT-Leish RDT and KD-POC showed the high LR+ (28.62; 95%CI: 9.40–87.16 and 27.64; 95%CI: 9.07–84.23, respectively) and the low LR- (0.06; 95%CI: 0.03–0.12 and 0.09; 95%CI: 0.05–0.16, respectively), which are in accordance to meta-analysis [40].

Concerning ELISA and IFA, we obtained unsatisfactory performance for the diagnosis of VL compared to Kalazar Detect and IT-Leish, as found by other studies [41]. ELISA demonstrated a poor specificity, ranging from 38.5–71.4% (56.0% in the total of samples), resulting in a low LR+ (2.15; 95%CI: 1.70–2.72) and a low accuracy (78.1%; 95%CI: 72.0–83.5). Other authors also obtained low specificity using total antigen-based ELISA [40–42]. IFA showed low accuracy (84.6%; 95%CI: 79.1–89.2) mostly due to the low sensitivity, ranging from 60.0–85.2% (80.6% in the total of samples), corroborating results obtained by other authors [31,40,41,43]

The variable sensitivity of RDT depending on the locality may be attributable to the parasite diversity. A study using multi-locus microsatellite typing revealed the presence of 67 genotypes of *L*. *infantum* in 162 strains from 17 Brazilian states and 11 from one locality in Paraguay. Clustering analysis detected the existence of three groups (POP1, POP2 and POP3). POP1 was observed in 17 of the studied localities (16 Brazilian states and Paraguay), and in some areas, it was markedly predominant. POP2 was predominant only in Mato Grosso. POP3 was observed mainly in Mato Grosso do Sul [44].

In São Paulo State, Brazil, multi-locus microsatellite typing analysis defined 33 different genotypes of *L*. *infantum* in 112 samples collected from dogs with visceral leishmaniasis: 67 from the northwestern region and 29 from the southeastern region. Authors correlated the results with 16 samples from Mato Grosso do Sul State, which borders the northwestern region. They found two main genetic clusters circulating in SP with strong genetic differentiation (POP-A and POP-B). POP-A was composed of 73.13% of the northwestern region (sub-POP-A1) and 89.66% of the southeastern region (sub-POP-A2) samples. POP-B was composed of 10.34% of the southeastern region and 26.87% of the northwestern region samples and 93.75% of MS parasite genotypes were in POP-B. The authors suggested that samples from POP-B in the northwestern region might be due to the expansion and dissemination of *L*. *infantum* from MS. In the southeastern region, infected dogs introduced a new *L*. *infantum* population, probably from other Brazilian regions or by imported dogs from other countries [45].

Bhattacharyya and collaborators [46] analyzed *L*. *donovani* kinesin polymorphisms and the divergence between East Africa and South Asia, in comparison with *L*. *infantum* (*L*. *chagasi*) rK39 (used in Kalazar Detect and IT-Leish). The authors showed a significant genetic diversity in coding sequences of rK39 homologs among strains in East Africa, between East Africa and South Asia and between East Africa and rK39 antigen. This genetic diversity could provide an explanation for the different performances of rK39- based RDT across regions.

rK39-RDT represents an important advance in the diagnosis of VL due to its ease of use, not requiring equipment and can be carried out at the POC in remote areas. Present results showing similar performance using whole blood samples is an important additional attribute for its use at the POC. Nevertheless, its lower sensitivity in some localities and coinfected patients raises a substantial concern on the routine use of these products for the diagnosis of VL.

One limitation of our study was the small number of controls with possible cross-reactivity, although the ELISA test showed false positive results with several of the samples from other diseases and RDT did not. Other limitation was the small number of coinfected VL/aids patients; nevertheless, the coinfected presented a significant lower sensitivity in relation to VL patients with all tests but IFA. Another limitation, that preclude the determination of predictive values was that the samples were not selected randomly, but by the previous diagnosis; nevertheless, the determination of LR overcome this limitation.

## Supporting information

S1 Fig Flow diagram for reporting the evaluation of Kalazar Detect using whole blood, serum and oral fluid from patients and controls, performed at the point of care. n–number of samples. ND–not done. VL–visceral leishmaniasis. DAT–direct agglutination test.
(DOCX)

S2 Fig Standardization of rapid diagnostic test reading. Test line intensities considered positive were scored as strong positive (3), positive (2), weak positive (1) and faint (0.5). The absence of test line was recorded as negative (0). C–control line. T–test line.
(TIF)

S3 Fig Flow diagram for reporting the comparison of Kalazar Detect with IT-Leish, ELISA and IFA serum from patients and controls. n–number of samples.
(DOCX)

S1 Table Diagnostic accuracy of “Kalazar Detect™ Rapid Test, Whole Blood” performed at the point of care, using oral fluid, serum and whole blood samples from VL patients, asymptomatic and potential cross-reactive controls, according to the collection site. n–number of samples. TP–true positive. FN–false positive. TN–true negative. FP–false positive. LR+–Positive likelihood ratio. LR-–Negative likelihood ratio. NC–not calculated. *–Samples from Sao Paulo (n = 15) and from VL/aids coinfected patients (n = 20) were not considered in this analysis.
(DOCX)

S2 Table Reader to reader agreement of Kalazar Detect performed at the point of care, using whole blood and serum samples. n–number of samples.
(DOCX)

S3 Table Lot to lot agreement of Kalazar Detect performed at the point of care, using whole blood and serum samples. n–number of samples.
(DOCX)

S4 Table Diagnostic accuracy of serological tests performed in serum from VL patients, asymptomatic and potential cross-reactive controls, according to the collection site. n–number of samples. TP–true positive. FN–false positive. TN–true negative. FP–false positive. LR+–Positive likelihood ratio. LR-–Negative likelihood ratio. NC–not calculated. KD-POC = Kalazar Detect performed at point of care. KD-IMT–Kalazar Detect processed at IMT. IT-Leish–rK39–RDT. IFA–*L*. *major*-like based Indirect immunofluorescence assay. ELISA–*L*. *major*-like based Enzyme-linked immunosorbent assay. *–Samples from Sao Paulo (n = 15) and from VL/aids coinfected patients (n = 20) were not considered in this analysis.
(DOCX)

S5 Table Sensitivity (%) and 95% confidence intervals (95% CI) of Kalazar Detect performed at the point of care (POC), and IT-Leish and other tests performed at IMT, in serum samples collected from patients with VL and VL/aids, according to the locality. n–number of samples. KD-POC–Kalazar Detect performed at the point of care. KD-IMT–Kalazar Detect processed at IMT. IT-Leish–rK39 –RDT. IFA–*L*. *major*-like based Indirect immunofluorescence assay. ELISA–*L*. *major*-like based Enzyme-linked immunosorbent assay. a–p = 0.0155, b–p = 0.0046, c–p = 0.0007, d–p = 0.0474, e–p = 0.0187, f–p = 0.0480, g–p = 0.0211 (Fisher’s exact test) in relation to VL.
(DOCX)

S6 Table Specificity (%) and 95% confidence intervals (95% CI) of IFA and ELISA performed at IMT, in serum samples from potential cross-reactive controls, according to the collection site. n–number of samples. IFA–*L*. *major*-like Indirect immunofluorescence assay. ELISA–*L*. *major*-like based Enzyme-linked immunosorbent assay.
(DOCX)

S1 Dataset Raw data obtained by the different tests and fluids applied to the various groups of samples studied. KD-POC–Kalazar Detect performed at the point of care. KD-IMT–Kalazar Detect processed at IMT. OF–oral fluid. SE–serum–WB–whole blood. DAT–direct agglutination test. IT-Leish–rK39 –RDT. IFA–*L*. *major*-like based indirect immunofluorescence assay. ELISA–*L*. *major*-like based enzyme-linked immunosorbent assay.
(XLSX)

S1 Checklist STARD checklist. (DOCX)

## References

[1] WHO. World Health Organization. Leishmaniasis: World Health Organization; 2019 [30/05/2019]. Available from: https://www.who.int/leishmaniasis/visceral_leishmaniasis/en/

[2] Srivastava P, Dayama A, Mehrotra S, Sundar S. Diagnosis of visceral leishmaniasis. Transactions of the Royal Society of Tropical Medicine and Hygiene. 2011 1;105(1):1–6. 10.1016/j.trstmh.2010.09.006 . Pubmed Central PMCID: PMC2999003. Epub 2010/11/16. eng. 21074233 PMC2999003

[3] Lindoso JAL, Costa JML, Queiroz IT, Goto H. Review of the current treatments for leishmaniases. Research and reports in tropical medicine. 2012;3:69–77. 10.2147/RRTM.S24764 . eng. 30890869 PMC6065590

[4] Alvar J, Canavate C, Molina R, Moreno J, Nieto J. Canine leishmaniasis. Adv Parasitol. 2004;57:1–88. 10.1016/S0065-308X(04)57001-X . Epub 2004/10/27. eng. 15504537

[5] WHO. World Health Organization. Leishmaniasis: World Health Organization; 2019 [29/05/2019]. Available from: http://www.who.int/news-room/fact-sheets/detail/leishmaniasis

[6] Brasil. Leishmaniose Visceral: o que é, causas, sintomas, tratamento, diagnóstico e prevenção 2019 [30/05/2019]. Available from: http://portalms.saude.gov.br/saude-de-a-z/leishmaniose-visceral

[7] Brasil. Casos confirmados de Leishmaniose Visceral, Brasil, Grandes Regiões e Unidades Federadas 2019 [30/05/2019]. Available from: http://portalarquivos2.saude.gov.br/images/pdf/2018/novembro/12/LV-Casos.pdf

[8] Brasil. Letalidade de Leishmaniose Visceral. Brasil, Grandes Regiões e Unidades Federadas. 2000 a 2017. 2019 [30/05/2019]. Available from: http://portalarquivos2.saude.gov.br/images/pdf/2018/novembro/12/LV-Letalidade.pdf

[9] Herwaldt BL. Leishmaniasis. Lancet (London, England). 1999 Oct 2;354(9185):1191–9. PubMed PMID: 10513726. Epub 1999/10/08. eng. 10.1016/S0140-6736(98)10178-2 10513726

[10] Cunningham J, Hasker E, Das P, El Safi S, Goto H, Mondal D, et al A global comparative evaluation of commercial immunochromatographic rapid diagnostic tests for visceral leishmaniasis. Clin Infect Dis. 2012 11 15;55(10):1312–9. 10.1093/cid/cis716 . Pubmed Central PMCID: PMC3478143. Epub 2012/09/04. eng. 22942208 PMC3478143

[11] Burns JM Jr., Shreffler WG, Benson DR, Ghalib HW, Badaro R, Reed SG. Molecular characterization of a kinesin-related antigen of Leishmania chagasi that detects specific antibody in African and American visceral leishmaniasis. Proc Natl Acad Sci U S A. 1993 1 15;90(2):775–9. 10.1073/pnas.90.2.775 . Pubmed Central PMCID: PMC45748. Epub 1993/01/15. eng. 8421715 PMC45748

[12] Sundar S, Reed SG, Singh VP, Kumar PC, Murray HW. Rapid accurate field diagnosis of Indian visceral leishmaniasis. Lancet (London, England). 1998 2 21;351(9102):563–5. 10.1016/S0140-6736(97)04350-X . Epub 1998/03/11. eng. 9492776

[13] Srividya G, Kulshrestha A, Singh R, Salotra P. Diagnosis of visceral leishmaniasis: developments over the last decade. Parasitol Res. 2012 3;110(3):1065–78. 10.1007/s00436-011-2680-1 . Epub 2011/11/09. eng. 22065060

[14] Chappuis F, Rijal S, Jha UK, Desjeux P, Karki BM, Koirala S, et al Field validity, reproducibility and feasibility of diagnostic tests for visceral leishmaniasis in rural Nepal. Trop Med Int Health. 2006 1;11(1):31–40. 10.1111/j.1365-3156.2005.01533.x . Epub 2006/01/10. eng. 16398753

[15] Singh DP, Goyal RK, Singh RK, Sundar S, Mohapatra TM. In search of an ideal test for diagnosis and prognosis of kala-azar. J Health Popul Nutr. 2010 6;28(3):281–5. 10.3329/jhpn.v28i3.5557 . Pubmed Central PMCID: PMC2980893. Epub 2010/07/20. eng. 20635639 PMC2980893

[16] Ritmeijer K, Melaku Y, Mueller M, Kipngetich S, O'Keeffe C, Davidson RN. Evaluation of a new recombinant K39 rapid diagnostic test for Sudanese visceral leishmaniasis. The American journal of tropical medicine and hygiene. 2006 1;74(1):76–80. . Epub 2006/01/13. eng. 16407349

[17] Chappuis F, Mueller Y, Nguimfack A, Rwakimari JB, Couffignal S, Boelaert M, et al Diagnostic accuracy of two rK39 antigen-based dipsticks and the formol gel test for rapid diagnosis of visceral leishmaniasis in northeastern Uganda. J Clin Microbiol. 2005 12;43(12):5973–7. 10.1128/JCM.43.12.5973-5977.2005 . Pubmed Central PMCID: PMC1317204. Epub 2005/12/08. eng. 16333084 PMC1317204

[18] Boelaert M, Bhattacharya S, Chappuis F, ois, El Safi S, Hailu A, et al Evaluation of Rapid Diagnostic Tests: Visceral Leishmaniasis. Nat Rev Microbiol. 2007 11/01;5:S30–S9.

[19] Banoo S, Bell D, Bossuyt P, Herring A, Mabey D, Poole F, et al Evaluation of diagnostic tests for infectious diseases: general principles. Nature reviews Microbiology. 2006 9;4(9 Suppl):S21–31. 10.1038/nrmicro1523 . Epub 2006/10/13. eng. 17034069

[20] el Harith A, Kolk AH, Leeuwenburg J, Muigai R, Huigen E, Jelsma T, et al Improvement of a direct agglutination test for field studies of visceral leishmaniasis. J Clin Microbiol. 1988;26(7):1321–5. . eng. 3410946 10.1128/jcm.26.7.1321-1325.1988 PMC266601

[21] Guimaraes MC, Celeste BJ, Franco EL, Cuce LC, Belda W Jr. Evaluation of serological diagnostic indices for mucocutaneous leishmaniasis: immunofluorescence tests and enzyme-linked immunoassays for IgG, IgM and IgA antibodies. Bull World Health Organ. 1989;67(6):643–8. . Pubmed Central PMCID: PMC2491305. Epub 1989/01/01. eng. 2699277 PMC2491305

[22] Celeste BJ, Guimaraes MC, de Souza JM, Bergamaschi DP. Reproducibility of alkaline antigens of Leishmania major-like and Leishmania (V.) braziliensis evaluated by IgG-ELISA. Comparison of antigens added of a protein inhibitor (PMSF) or not. Rev Inst Med Trop Sao Paulo. 1998 Sep-Oct;40(5):287–90. 10.1590/s0036-46651998000500004 . Epub 1999/02/25. eng. 10030072

[23] Fleiss J, Levin B, Paik M. Statistical Methods for Rates and Proportions, Third Edition. John Wiley & Sons, Inc Hoboken, New Jersey; 2003.

[24] Landis JR, Koch GG. An application of hierarchical kappa-type statistics in the assessment of majority agreement among multiple observers. Biometrics. 1977 6;33(2):363–74. . Epub 1977/06/01. eng. 884196

[25] Simel DL, Samsa GP, Matchar DB. Likelihood ratios with confidence: sample size estimation for diagnostic test studies. Journal of clinical epidemiology. 1991;44(8):763–70. 10.1016/0895-4356(91)90128-v . Epub 1991/01/01. eng. 1941027

[26] Grimes DA, Schulz KF. Refining clinical diagnosis with likelihood ratios. Lancet (London, England). 2005 4 23–29;365(9469):1500–5. 10.1016/S0140-6736(05)66422-7 . Epub 2005/04/27. eng. 15850636

[27] Deeks JJ, Altman DG. Diagnostic tests 4: likelihood ratios. BMJ. 2004;329(7458):168–9. 10.1136/bmj.329.7458.168 . eng. 15258077 PMC478236

[28] Furukawa TA. SS, Bucher HC, Guya G. DIAGNOSTIC TESTS In: Gordon Guyatt MOM, Rennie Drummond, Cook Deborah J., editor. USERS’ GUIDES TO THE MEDICAL LITERATURE—ESSENTIALS OF EVIDENCE-BASED CLINICAL PRACTICE. Second ed2008 p. 195–222.

[29] Attia J. Moving beyond sensitivity and specificity: Using likelihood ratios to help interpret diagnostic tests. Australian Prescriber. 2002 11/30;26.

[30] Grimes DA, Schulz KF. Uses and abuses of screening tests. Lancet (London, England). 2002 3 9;359(9309):881–4. 10.1016/S0140-6736(02)07948-5 . Epub 2002/03/19. eng. 11897304

[31] Bangert M, Flores-Chavez MD, Llanes-Acevedo IP, Arcones C, Chicharro C, Garcia E, et al Validation of rK39 immunochromatographic test and direct agglutination test for the diagnosis of Mediterranean visceral leishmaniasis in Spain. PLoS Negl Trop Dis. 2018 3;12(3):e0006277 10.1371/journal.pntd.0006277 . Pubmed Central PMCID: PMC5849364. Epub 2018/03/02. eng. 29494596 PMC5849364

[32] da Silva MRB, Brandao NAA, Colovati M, de Sousa MMP, de Lima LC, Dorta ML, et al Performance of two immunochromatographic tests for diagnosis of visceral leishmaniasis in patients coinfected with HIV. Parasitol Res. 2018 2;117(2):419–27. 10.1007/s00436-017-5716-3 . Epub 2017/12/23. eng. 29270768

[33] Peruhype-Magalhaes V, Machado-de-Assis TS, Rabello A. Use of the Kala-Azar Detect(R) and IT-LEISH(R) rapid tests for the diagnosis of visceral leishmaniasis in Brazil. Memorias do Instituto Oswaldo Cruz. 2012 11;107(7):951–2. 10.1590/s0074-02762012000700019 . Epub 2012/11/14. eng. 23147155

[34] Vaish M, Singh OP, Chakravarty J, Sundar S. rK39 antigen for the diagnosis of visceral leishmaniasis by using human saliva. The American journal of tropical medicine and hygiene. 2012 4;86(4):598–600. 10.4269/ajtmh.2012.11-0127 . Pubmed Central PMCID: PMC3403768. Epub 2012/04/12. eng. 22492142 PMC3403768

[35] Mohapatra S, Samantaray JC, Ghosh A. A Comparative Study of Serum, Urine and Saliva Using rk39 Strip for the Diagnosis of Visceral Leishmaniasis. J Arthropod Borne Dis. 2016 3;10(1):87–91. . Pubmed Central PMCID: PMC4813396. Epub 2016/04/06. eng. 27047975 PMC4813396

[36] da Silva MR, Brandao NA, Dorta ML, Fatima RD, Costa DL, Costa CH, et al Evaluation of an rK39-based immunochromatographic test for the diagnosis of visceral leishmaniasis in human saliva. Trop Biomed. 2015 6;32(2):247–56. . Epub 2015/12/23. eng. 26691253

[37] Galai Y, Chabchoub N, Ben-Abid M, Ben-Abda I, Ben-Alaya-Bouafif N, Amri F, et al Diagnosis of mediterranean visceral leishmaniasis by detection of leishmania antibodies and leishmania DNA in oral fluid samples collected using an Oracol device. J Clin Microbiol. 2011 9;49(9):3150–3. 10.1128/JCM.00267-11 . Pubmed Central PMCID: PMC3165591. Epub 2011/07/01. eng. 21715587 PMC3165591

[38] van Griensven J, Diro E. Visceral Leishmaniasis: Recent Advances in Diagnostics and Treatment Regimens. Infect Dis Clin North Am. 2019 3;33(1):79–99. 10.1016/j.idc.2018.10.005 . Epub 2019/02/05. eng. 30712769

[39] Cota GF, de Sousa MR, de Freitas Nogueira BM, Gomes LI, Oliveira E, Assis TS, et al Comparison of parasitological, serological, and molecular tests for visceral leishmaniasis in HIV-infected patients: a cross-sectional delayed-type study. The American journal of tropical medicine and hygiene. 2013 9;89(3):570–7. 10.4269/ajtmh.13-0239 . Pubmed Central PMCID: PMC3771302. Epub 2013/07/10. eng. 23836568 PMC3771302

[40] Maia Z, Lirio M, Mistro S, Mendes CM, Mehta SR, Badaro R. Comparative study of rK39 Leishmania antigen for serodiagnosis of visceral leishmaniasis: systematic review with meta-analysis. PLoS Negl Trop Dis. 2012 1;6(1):e1484 10.1371/journal.pntd.0001484 . Pubmed Central PMCID: PMC3269412. Epub 2012/02/04. eng. 22303488 PMC3269412

[41] Freire ML, Machado de Assis T, Oliveira E, Moreira de Avelar D, Siqueira IC, Barral A, et al Performance of serological tests available in Brazil for the diagnosis of human visceral leishmaniasis. PLOS Neglected Tropical Diseases. 2019;13(7):e0007484 10.1371/journal.pntd.0007484 31318856 PMC6638734

[42] Pedras MJ, de Gouvea Viana L, de Oliveira EJ, Rabello A. Comparative evaluation of direct agglutination test, rK39 and soluble antigen ELISA and IFAT for the diagnosis of visceral leishmaniasis. Transactions of the Royal Society of Tropical Medicine and Hygiene. 2008 2;102(2):172–8. 10.1016/j.trstmh.2007.11.004 . Epub 2007/12/28. eng. 18160087

[43] Mikaeili F, Fakhar M, Sarkari B, Motazedian MH, Hatam G. Comparison of serological methods (ELISA, DAT and IFA) for diagnosis of visceral leishmaniasis utilizing an endemic strain. Iranian journal of immunology: IJI. 2007 6;4(2):116–21. doi: IJIv4i2A7 . Epub 2007/07/27. eng. 17652852 10.22034/iji.2007.17188

[44] Ferreira GE, dos Santos BN, Dorval ME, Ramos TP, Porrozzi R, Peixoto AA, et al The genetic structure of Leishmania infantum populations in Brazil and its possible association with the transmission cycle of visceral leishmaniasis. PLoS One. 2012;7(5):e36242 10.1371/journal.pone.0036242 . Pubmed Central PMCID: PMC3350531. Epub 2012/05/19. eng. 22606248 PMC3350531

[45] Motoie G, Ferreira GE, Cupolillo E, Canavez F, Pereira-Chioccola VL. Spatial distribution and population genetics of Leishmania infantum genotypes in Sao Paulo State, Brazil, employing multilocus microsatellite typing directly in dog infected tissues. Infect Genet Evol. 2013 8;18:48–59. 10.1016/j.meegid.2013.04.031 . Epub 2013/05/15. eng. 23665466

[46] Bhattacharyya T, Boelaert M, Miles MA. Comparison of visceral leishmaniasis diagnostic antigens in African and Asian Leishmania donovani reveals extensive diversity and region-specific polymorphisms. PLoS Negl Trop Dis. 2013;7(2):e2057 10.1371/journal.pntd.0002057 . Pubmed Central PMCID: PMC3585016. Epub 2013/03/08. eng. 23469296 PMC3585016

