# Correction: Performance of rK39-based immunochromatographic rapid diagnostic test for serodiagnosis of visceral leishmaniasis using whole blood, serum and oral fluid

**DOI:** 10.1371/journal.pone.0232727

**Published:** 2020-04-29

**Authors:** Maria Carmen Arroyo Sanchez, Beatriz Julieta Celeste, José Angelo Lauletta Lindoso, Mahyumi Fujimori, Roque Pacheco de Almeida, Carlos Magno Castelo Branco Fortaleza, Angelita Fernandes Druzian, Ana Priscila Freitas Lemos, Vanessa Campos Andrade de Melo, Anamaria Mello Miranda Paniago, Igor Thiago Queiroz, Hiro Goto

The following information is missing from the Funding statement: This study was also supported by the Coordenação de Aperfeiçoamento de Pessoal de Nível Superior—Brasil (CAPES)—Finance Code 001.
