# Supplementary material for: Performance of rK39-based immunochromatographic rapid diagnostic test for serodiagnosis of visceral leishmaniasis using whole blood, serum and oral fluid
Source: PLoS One. 2020 Apr 2;15(4):e0230610. doi: 10.1371/journal.pone.0230610 (PMC7117722; doi:10.1371/journal.pone.0230610)
Supplement: S1 Fig — n–number of samples. ND–not done. VL–visceral leishmaniasis. DAT–direct agglutination test. (DOCX) [file pone.0230610.s001.docx]

Excluded – n = 22

-No index test – n = 3

-No reference test – n = 13

-Control (HIV positive) – n = 6

Potentially eligible participants

n = 282

Excluded – n = 5

-Control (DAT positive) – n = 3

-VL (DAT negative/parasitology ND) – n = 2

Eligible participants

n = 260

Reference Standard

n = 255

DAT

Asymptomatic controls (AC) – n = 85

Other diseases (OD) – n = 22

Parasitology/DAT

VL patients – n = 128

VL/aids patients – n = 20

Index Test – Kalazar Detect

n = 255

Kalazar Detect (whole blood)

n = 255

Kalazar Detect (oral fluid)

n = 255

Kalazar Detect (serum)

n = 255

Positive Test

VL patients – n = 116

VL/aids patients – n = 14

AC + OD – n = 0

Positive Test

VL patients – n = 94

VL/aids patients – n = 9

AC + OD – n = 5

Positive Test

VL patients – n = 115

VL/aids patients – n = 14

AC + OD – n = 4

Negative Test

VL patients – n = 34

VL/aids patients – n = 11

AC + OD – n = 102

Negative Test

VL patients – n = 13

VL/aids patients – n = 6

AC + OD – n = 103

Negative Test

VL patients – n = 12

VL/aids patients – n = 6

AC + OD – n = 107

**S1 Figure. Flow diagram for reporting the evaluation of Kalazar Detect using whole blood, serum and oral fluid from patients and controls, performed at the point of care.** n – number of samples. ND – not done. VL – visceral leishmaniasis. DAT – direct agglutination test.
