# Supplementary figures and images for: Performance of rK39-based immunochromatographic rapid diagnostic test for serodiagnosis of visceral leishmaniasis using whole blood, serum and oral fluid
Source: PLoS One. 2020 Apr 2;15(4):e0230610. doi: 10.1371/journal.pone.0230610 (PMC7117722; doi:10.1371/journal.pone.0230610)

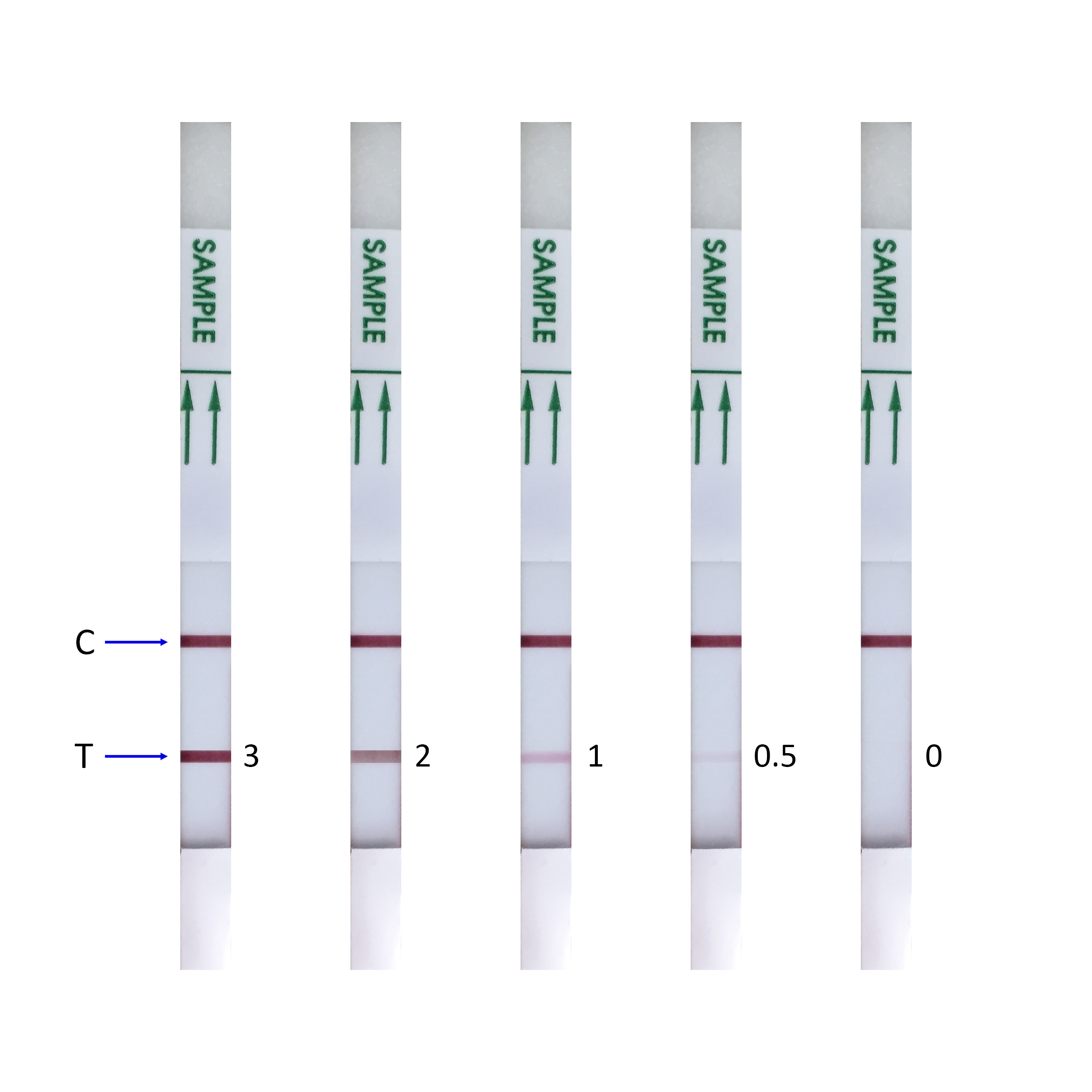

Supplement: S2 Fig — Test line intensities considered positive were scored as strong positive (3), positive (2), weak positive (1) and faint (0.5). The absence of test line was recorded as negative (0). C–control line. T–test line. (TIF) [file pone.0230610.s002.tif]
