# Supplementary material for: Performance of rK39-based immunochromatographic rapid diagnostic test for serodiagnosis of visceral leishmaniasis using whole blood, serum and oral fluid
Source: PLoS One. 2020 Apr 2;15(4):e0230610. doi: 10.1371/journal.pone.0230610 (PMC7117722; doi:10.1371/journal.pone.0230610)
Supplement: S3 Fig — n–number of samples. (DOCX) [file pone.0230610.s003.docx]

Eligible participants

n = 255

Serum samples not sent to IMT/FMUSP – n = 5

Serum samples sent to IMT/FMUSP

n = 250

Reference Standard

n = 250

DAT

Asymptomatic controls – n = 84

Other diseases – n = 22

Parasitology/DAT

VL patients – n = 124

VL/aids patients – n = 20

Kalazar Detect – POC

n = 250

IT-Leish

n = 250

Index Test – Kalazar Detect

n = 250

ELISA

n = 250

IFA

n = 250

Negative Test

VL patients – n = 24

VL/aids patients – n = 5

AC + OD – n = 93

Positive Test

VL patients – n = 100

VL/aids patients – n = 15

AC + OD – n = 13

Positive Test

VL patients – n = 117

VL/aids patients – n = 13

AC + OD – n = 3

Positive Test

VL patients – n = 109

VL/aids patients – n = 12

AC + OD – n = 7

Positive Test

VL patients – n = 113

VL/aids patients – n = 14

AC + OD – n = 3

Positive Test

VL patients – n = 117

VL/aids patients – n = 16

AC + OD – n = 51

Negative Test

VL patients – n = 15

VL/aids patients – n = 8

AC + OD – n = 99

Negative Test

VL patients – n = 7

VL/aids patients – n = 7

AC + OD – n = 103

Negative Test

VL patients – n = 11

VL/aids patients – n = 6

AC + OD – n = 103

Negative Test

VL patients – n = 7

VL/aids patients – n = 4

AC + OD – n = 55

**S2 Figure. Flow diagram for reporting the comparison of Kalazar Detect with IT-Leish, ELISA and IFA serum from patients and controls.**
