# Supplementary material for: Performance of rK39-based immunochromatographic rapid diagnostic test for serodiagnosis of visceral leishmaniasis using whole blood, serum and oral fluid
Source: PLoS One. 2020 Apr 2;15(4):e0230610. doi: 10.1371/journal.pone.0230610 (PMC7117722; doi:10.1371/journal.pone.0230610)
Supplement: S1 Table — n–number of samples. TP–true positive. FN–false positive. TN–true negative. FP–false positive. LR+–Positive likelihood ratio. LR-–Negative likelihood ratio. NC–not calculated. *–Samples from Sao Paulo (n = 15) and from VL/aids coinfected patients (n = 20) were not considered in this analysis. (DOCX) [file pone.0230610.s004.docx]

**S1 Table. Diagnostic accuracy of Kalazar Detect™ Rapid Test, Whole Blood performed at the point of care, using oral fluid, serum and whole blood samples from VL patients, asymptomatic and potential cross-reactive controls, according to the collection site**

| **Locality (n)** | **Fluid** | **Number of individuals** | | | | | **Diagnostic accuracy (95% CI)** | | | | |
| --- | --- | --- | --- | --- | --- | --- | --- | --- | --- | --- | --- |
|  |  | **TP** | **FN** | **TN** | **FP** |  | **Sensitivity %** | **Specificity %** | **LR +** | **LR -** | **Accuracy %** |
| **Campo Grande**  **(65)** | **Oral fluid** | 24 | 3 | 35 | 3 |  | 88.9 (71.9-96.1) | 92.1 (79.2-97.3) | 11.26 (3.77-33.63) | 0.12 (0.04-0.35) | 90.8 (81.0-96.5) |
|  | **Serum** | 26 | 1 | 36 | 2 |  | 96.3 (81.7-99.3) | 94.7 (82.7-98.5) | 18.30 (4.74-70.65) | 0.04 (0.01-0.27) | 95.4 (87.1-99.0) |
|  | **Whole blood** | 26 | 1 | 38 | 0 |  | 96.3 (81.7-99.3) | 100.0 (90.8-100.0) | NC | 0.04 (0.01-0.25) | 98.5 (91.7-100.0) |
| **Bauru (23)** | **Oral fluid** | 13 | 3 | 7 | 0 |  | 81.2 (57.0-93.4) | 100.0 (64.6-100.0) | NC | 0.19 (0.07-0.52) | 87.0 (66.4-97.2) |
|  | **Serum** | 15 | 1 | 7 | 0 |  | 93.7 (71.7-98.9) | 100.0 (64.6-100.0) | NC | 0.06 (0.01-0.42) | 95.6 (78.0- 99.9) |
|  | **Whole blood** | 15 | 1 | 7 | 0 |  | 93.7 (71.7-98.9) | 100.0 (64.6-100.0) | NC | 0.06 (0.01-0.42) | 95.6 (78.0- 99.9) |
| **Aracaju (69)** | **Oral fluid** | 44 | 11 | 13 | 1 |  | 80.0 (67.6-88.4) | 92.9 (68.5-98.7) | 11.20 (1.69-74.38) | 0.22 (0.12-0.37) | 82.6 (71.6-90.7) |
|  | **Serum** | 50 | 5 | 12 | 2 |  | 90.9 (80.4-96.0) | 85.7 (60.1-96.0) | 6.36 (1.76-23.02) | 0.11 (0.04-0.25) | 89.9 (80.2-95.8) |
|  | **Whole blood** | 51 | 4 | 14 | 0 |  | 92.7 (82.7-97.1) | 100.0 (78.5-100.0) | NC | 0.07 (0.03-0.19) | 94.2 (85.8-98.4) |
| **Natal (63)** | **Oral fluid** | 13 | 17 | 33 | 0 |  | 43.3 (27.4-60.8) | 100.0 (89.6-100.0) | NC | 0.57 (0.41-0.77) | 73.0 (60.3-83.4) |
|  | **Serum** | 24 | 6 | 33 | 0 |  | 80.0 (62.7-90.5) | 100.0 (89.6-100.0) | NC | 0.20 (0.10-0.41) | 90.5 (80.4-96.4) |
|  | **Whole blood** | 24 | 6 | 33 | 0 |  | 80.0 (62.7-90.5) | 100.0 (89.6-100.0) | NC | 0.20 (0.10-0.41) | 90.5 (80.4-96.4) |
| **Total (220) *** | **Oral fluid** | 94 | 34 | 88 | 4 |  | 73.4 (64.9-80.8) | 95.6 (89.2-98.8) | 16.89 (6.44-44.29) | 0.28 (0.21-0.37) | 82.7 (77.1-87.5) |
|  | **Serum** | 115 | 13 | 88 | 4 |  | 89.8 (83.3-94.5) | 95.6 (89.2-98.8) | 20.66 (7.91-53.98) | 0.11 (0.06-0.18) | 92.3 (87.9-95.4) |
|  | **Whole blood** | 116 | 12 | 92 | 0 |  | 90.6 (84.2-95.1) | 100.0 (96.1-100.0) | NC | 0.09 (0.05-0.16) | 94.5 (90.7-97.1) |

n – number of samples. TP – true positive. FN – false positive. TN – true negative. FP – false positive.

LR+ – Positive likelihood ratio. LR- – Negative likelihood ratio. NC – not calculated.

* – Samples from Sao Paulo (n=15) and from VL/aids coinfected patients (n=20) were not considered in this analysis.
