# Supplementary material for: Performance of rK39-based immunochromatographic rapid diagnostic test for serodiagnosis of visceral leishmaniasis using whole blood, serum and oral fluid
Source: PLoS One. 2020 Apr 2;15(4):e0230610. doi: 10.1371/journal.pone.0230610 (PMC7117722; doi:10.1371/journal.pone.0230610)
Supplement: S3 Table — n–number of samples. (DOCX) [file pone.0230610.s006.docx]

**S3 Table.** **Lot to lot agreement of Kalazar Detect performed at the point of care, using whole blood and serum samples**

| **Locality (n)** | **Kappa (95% CI)** | |
| --- | --- | --- |
|  | **Whole Blood** | **Serum** |
| **Campo Grande (26)** | 1.00 (1.00-1.00) | 0.91 (0.73-1.00) |
| **Bauru (7)** | 1.00 (1.00-1.00) | 1.00 (1.00-1.00) |
| **Natal (34)** | 1.00 (1.00-1.00) | 1.00 (1.00-1.00) |
| **Total (67)** | 1.00 (1.00-1.00) | 0.97 (0.90-1.00) |

n = number of samples
