# Supplementary material for: Performance of rK39-based immunochromatographic rapid diagnostic test for serodiagnosis of visceral leishmaniasis using whole blood, serum and oral fluid
Source: PLoS One. 2020 Apr 2;15(4):e0230610. doi: 10.1371/journal.pone.0230610 (PMC7117722; doi:10.1371/journal.pone.0230610)
Supplement: S4 Table — n–number of samples. TP–true positive. FN–false positive. TN–true negative. FP–false positive. LR+–Positive likelihood ratio. LR-–Negative likelihood ratio. NC–not calculated. KD-POC = Kalazar Detect performed at point of care. KD-IMT–Kalazar Detect processed at IMT. IT-Leish–rK39–RDT. IFA–L. major-like based Indirect immunofluorescence assay. ELISA–L. major-like based Enzyme-linked immunosorbent assay. *–Samples from Sao Paulo (n = 15) and from VL/aids coinfected patients (n = 20) were not considered in this analysis. (DOCX) [file pone.0230610.s007.docx]

**S4 Table. Diagnostic accuracy of serological tests performed in serum from VL patients, asymptomatic and potential cross-reactive controls, according to the collection site**

| **Locality (n)** | **Test** | **Number of individuals** | | | | | **Diagnostic accuracy (95% CI)** | | | | |
| --- | --- | --- | --- | --- | --- | --- | --- | --- | --- | --- | --- |
|  |  | **TP** | **FN** | **TN** | **FP** |  | **Sensitivity %** | **Specificity %** | **LR +** | **LR -** | **Accuracy %** |
| **Campo Grande (65)** | **KD POC** | 26 | 1 | 36 | 2 |  | 96.3 (81.7-99.3) | 94.7 (82.7-98.5) | 18.30 (4.74-70.65) | 0.04 (0.01-0.27) | 95.4 (87.1-99.0) |
|  | **KD IMT** | 26 | 1 | 34 | 4 |  | 96.3 (81.7-99.3) | 89.5 (75.9-95.8) | 9.15 (3.61-23.18) | 0.04 (0.01-0.28) | 92.3 (82.9-97.5) |
|  | **IT Leish** | 26 | 1 | 37 | 1 |  | 96.3 (81.7-99.3) | 97.4 (86.5-99.5) | 36.59 (5.28-253.48) | 0.04 (0.01-0.26) | 96.9 (89.3-99.6) |
|  | **IFA** | 23 | 4 | 37 | 1 |  | 85.2 (67.5-94.4) | 97.4 (86.5-99.5) | 32.37 (4.65-225.35) | 0.15 (0.06-0.38) | 92.3 (82.9-97.5) |
|  | **ELISA** | 26 | 1 | 21 | 17 |  | 96.3 (81.7-99.3) | 55.3 (39.7-69.8) | 2.15 (1.50-3.09) | 0.07 (0.01-0.47) | 72.3 (59.8-82.7) |
| **Bauru (22)** | **KD POC** | 14 | 1 | 7 | 0 |  | 93.3 (70.2-98.8) | 100.0 (64.6-100.0) | NC | 0.07 (0.01-0.44) | 95.4 (77.2-99.9) |
|  | **KD IMT** | 14 | 1 | 7 | 0 |  | 93.3 (70.2-98.8) | 100.0 (64.6-100.0) | NC | 0.07 (0.01-0.44) | 95.4 (77.2-99.9) |
|  | **IT Leish** | 14 | 1 | 7 | 0 |  | 93.3 (70.2-98.8) | 100.0 (64.6-100.0) | NC | 0.07 (0.01-0.44) | 95.4 (77.2-99.9) |
|  | **IFA** | 9 | 6 | 7 | 0 |  | 60.0 (35.7-80.2) | 100.0 (64.6-100.0) | NC | 0.40 (0.22-0.74) | 72.7 (49.8-89.3) |
|  | **ELISA** | 14 | 1 | 5 | 2 |  | 93.3 (70.2-98.8) | 71.4 (35.9-91.8) | 1.31 (0.80-2.13) | 0.23 (0.03-2.16) | 72.7 (49.8-89.3) |
| **Aracaju (65)** | **KD POC** | 49 | 3 | 12 | 1 |  | 94.2 (84.4-98.0) | 92.3 (63.7-98.6) | 12.25 (1.86-80.63) | 0.06 (0.02-0.19) | 93.8 (85.0-98.3) |
|  | **KD IMT** | 45 | 7 | 11 | 2 |  | 86.5 (74.7-93.3) | 84.6 (57.8-95.7) | 5.62 (1.56-20.22) | 0.16 (0.08-0.33) | 86.1 (75.3-93.5) |
|  | **IT Leish** | 51 | 1 | 11 | 2 |  | 98.1 (89.5-99.6) | 84.6 (57.8-95.7) | 6.37 (1.78-22.82) | 0.02 (0.00-0.16) | 95.4 (87.1-99.0) |
|  | **IFA** | 43 | 9 | 10 | 3 |  | 82.7 (70.2-90.6) | 76.9 (49.7-91.8) | 3.58 (1.32-9.74) | 0.23 (0.12-0.44) | 81.5 (70.0-90.1) |
|  | **ELISA** | 51 | 1 | 5 | 8 |  | 98.1 (89.9-99.7) | 38.5 (17.7-64.5) | 1.59 (1.04-2.45) | 0.05 (0.01-0.39) | 86.1 (75.3-93.5) |
| **Natal (63)** | **KD POC** | 24 | 6 | 33 | 0 |  | 80.0 (62.7-90.5) | 100.0 (89.6-100.0) | NC | 0.20 (0.10-0.41) | 90.5 (80.4-96.4) |
|  | **KD IMT** | 24 | 6 | 32 | 1 |  | 80.0 (62.7-90.5) | 97.0 (84.7-99.5) | 26.40 (3.80-183.41) | 0.21 (0.10-0.42) | 88.9 (78.4-95.4) |
|  | **IT Leish** | 26 | 4 | 33 | 0 |  | 86.7 (70.3-94.7) | 100.0 (89.6-100.0) | NC | 0.13 (0.05-0.33) | 93.6 (84.5-98.2) |
|  | **IFA** | 25 | 5 | 28 | 5 |  | 83.3 (66.4-92.7) | 84.8 (69.1-93.3) | 5.50 (2.41-12.53) | 0.20 (0.09-0.44) | 84.1 (72.7-92.1) |
|  | **ELISA** | 26 | 4 | 20 | 13 |  | 86.7 (70.3-94.7) | 60.6 (43.7-75.3) | 2.20 (1.41-3.44) | 0.22 (0.08-0.57) | 73.0 (60.3-83.4) |
| **Total (215)** | **KD POC** | 113 | 11 | 88 | 3 |  | 91.1 (84.7-95.5) | 96.7 (90.7-99.3) | 27.64 (9.07-84.23) | 0.09 (0.05-0.16) | 93.5 (89.3-96.4) |
|  | **KD IMT** | 109 | 15 | 84 | 7 |  | 87.9 (80.8-93.1) | 92.3 (84.8-96.8) | 11.43 (5.59-23.35) | 0.13 (0.08-0.21) | 89.8 (84.9-93.5) |
|  | **IT Leish** | 117 | 7 | 88 | 3 |  | 94.3 (88.7-97.7) | 96.7 (90.7-99.3) | 28.62 (9.40-87.16) | 0.06 (0.03-0.12) | 95.3 (91.6-97.7) |
|  | **IFA** | 100 | 24 | 82 | 9 |  | 80.6 (72.6-87.2) | 90.1 (82.0-95.4) | 8.15 (4.36-15.25) | 0.21 (0.15-0.31) | 84.6 (79.1-89.2) |
|  | **ELISA** | 117 | 7 | 51 | 40 |  | 94.3 (88.7-97.7) | 56.0 (45.2-66.4) | 2.15 (1.70-2.72) | 0.10 (0.05-0.21) | 78.1 (72.0-83.5) |

n – number of samples. TP – true positive. FN – false positive. TN – true negative. FP – false positive.

LR+ – Positive likelihood ratio. LR- – Negative likelihood ratio. NC – not calculated. KD-POC = Kalazar Detect performed at point of care. KD-IMT – Kalazar Detect processed at IMT. IT-Leish – rK39–RDT. IFA – *L. major*-like based Indirect immunofluorescence assay. ELISA – *L. major*-like based Enzyme-linked immunosorbent assay.

* – Samples from Sao Paulo (n=15) and from VL/aids coinfected patients (n=20) were not considered in this analysis.
