# Supplementary material for: Performance of rK39-based immunochromatographic rapid diagnostic test for serodiagnosis of visceral leishmaniasis using whole blood, serum and oral fluid
Source: PLoS One. 2020 Apr 2;15(4):e0230610. doi: 10.1371/journal.pone.0230610 (PMC7117722; doi:10.1371/journal.pone.0230610)
Supplement: S5 Table — n–number of samples. KD-POC–Kalazar Detect performed at the point of care. KD-IMT–Kalazar Detect processed at IMT. IT-Leish–rK39 –RDT. IFA–L. major-like based Indirect immunofluorescence assay. ELISA–L. major-like based Enzyme-linked immunosorbent assay. a–p = 0.0155, b–p = 0.0046, c–p = 0.0007, d–p = 0.0474, e–p = 0.0187, f–p = 0.0480, g–p = 0.0211 (Fisher’s exact test) in relation to VL. (DOCX) [file pone.0230610.s008.docx]

**S5 Table. Sensitivity (%) and 95% confidence intervals (95% CI) of Kalazar Detect performed at the point of care (POC) and IT-Leish and other tests performed at IMT, in serum samples collected from patients with VL and VL/aids, according to the locality**

| **Locality** | **Patients** | **Sensitivity % (n)**  **95% CI** | | | | |
| --- | --- | --- | --- | --- | --- | --- |
|  |  | **KD-POC** | **KD-IMT** | **IT-Leish** | **IFA** | **ELISA** |
| **Campo Grande** | **VL** | 96.3 (26)  81.7-99.3 | 96.3 (26)  81.7-99.3 | 96.3 (26)  81.7-99.3 | 85.2 (23)  67.5-94.4 | 96.3 (26)  81.7-99.3 |
|  | **VL/aids** | 81.8 (9)  79.2-97.3 | 63.6 (7) *e*  35.4-84.8 | 72.7 (8)  43.4-90.3 | 72.7 (8)  43.4-90.3 | 81.8 (9)  79.2-97.3 |
| **Bauru** | **VL** | 93.3 (14)  70.2-98.8 | 93.3 (14)  70.2-98.8 | 93.3 (14)  70.2-98.8 | 60.0 (9)  35.7-80.2 | 93.3 (14)  70.2-98.8 |
|  | **VL/aids** | 80.0 (4)  37.6-96.4 | 80.0 (4)  37.6-96.4 | 80.0 (4)  37.6-96.4 | 80.0 (4)  37.6-96.4 | 80.0 (4)  37.6-96.4 |
| **Natal** | **VL** | 80.0 (24)  62.7-90.5 | 80.0 (24)  62.7-90.5 | 86.7 (26)  70.3-94.7 | 83.3 (25)  66.4-92.7 | 86.7 (26)  70.3-94.7 |
|  | **VL/aids** | 25.0 (1) *f*  4.56-70.0 | 25.0 (1) *f*  4.6-69.9 | 25.0 (1) *g*  4.6-69.9 | 75.0 (3)  30.1-95.4 | 75.0 (3)  30.1-95.4 |
| **Total** | **VL** | **91.1 (113)**  **84.8-95.0** | **87.9 (109)**  **81.0-92.5** | **94.4 (117)**  **88.8-97.2** | **80.6 (100)**  **72.8-86.6** | **94.4 (117)**  **88.8-97.2** |
|  | **VL/aids** | **70.0 (14) *a***  **48.1-85.5** | **60.0 (12) *b***  **38.7-78.1** | **65.0 (13) *c***  **43.3-81.9** | **75.0 (15)**  **53.1-88.8** | **80.0 (16) *d***  **58.4-91.9** |

n – number of samples. KD-POC – Kalazar Detect performed at the point of care. KD-IMT – Kalazar Detect processed at IMT. IT-Leish – rK39 –RDT. IFA – *L. major*-like based Indirect immunofluorescence assay. ELISA – *L. major*-like based Enzyme-linked immunosorbent assay. *a* – p=0.0155, *b* – p=0.0046, *c* – p=0.0007, *d* – p=0.0474, *e* – p=0.0187, *f* – p=0.0480, *g* – p=0.0211 (Fisher’s exact test) in relation to VL.
