# Supplementary material for: Performance of rK39-based immunochromatographic rapid diagnostic test for serodiagnosis of visceral leishmaniasis using whole blood, serum and oral fluid
Source: PLoS One. 2020 Apr 2;15(4):e0230610. doi: 10.1371/journal.pone.0230610 (PMC7117722; doi:10.1371/journal.pone.0230610)
Supplement: S6 Table — n–number of samples. IFA–L. major-like Indirect immunofluorescence assay. ELISA–L. major-like based Enzyme-linked immunosorbent assay. (DOCX) [file pone.0230610.s009.docx]

**S6 Table.** **Specificity (%) and 95% confidence intervals (95% CI) of IFA and ELISA performed at IMT, in serum samples from potential cross-reactive controls, according to the collection site**

| **Locality** | **Disease (n)** | IFA (n) | ELISA (n) |
| --- | --- | --- | --- |
| **Campo Grande** | **Paracoccidioidomycosis (7)** | 85.7 (6)  48.7-97.4 | 14.3 (1)  2.6-51.3 |
| **Sao Paulo** | **Tegumentar leishmaniasis (7)** | 100.0 (7)  64.6-100.0 | 28.6 (2)  8.2-64.1 |
|  | **Chagas Disease (6)** | 50.0 (3)  18.8-81.2 | 0.0 (0)  0.0-39.0 |
|  | **Tuberculosis 92)** | 100.0 (2)  34.2-100.0 | 100.0 (2)  34.2-100.0 |
| **Total** | | 81.2 (18)  61.5-92.7 | 22.7 (5) 10.1-43.4 |

n = number of samples

IFA – *L. major*-like Indirect immunofluorescence assay

ELISA – *L. major*-like based Enzyme-linked immunosorbent assay
